# 

**Published:** 1866-09

**Authors:** 


### Dental Register.

J. TAFT,

EDITOR AND PUBLISHER.

SEPTEMBER, 1866.

MONTHLY:

THREE DOLLARS PER ANNUM, IN ADVANCE.

CINCINNATI:
WRIGHTSON & CO., Printers, 167 WALNUT STREET.

*J. & C. R. TAFT, Dentists, 238 West Fourth St.*

